# Focal Dystonia in a Patient With Young-Onset Parkinsonism: A Diagnostic Challenge

**DOI:** 10.7759/cureus.90203

**Published:** 2025-08-16

**Authors:** Ahmer A Longi, Pushparaja Shetty, Misbah Fazlani, Mohammed Alnims, Nauman Ali

**Affiliations:** 1 Internal Medicine, Mediclinic Welcare Hospital, Dubai, ARE; 2 Neurology, Mediclinic Welcare Hospital, Dubai, ARE

**Keywords:** focal dystonia, oropharyngeal dystonia, parkinsonism, tongue dystonia, young-onset parkinson’s disease

## Abstract

Oropharyngeal dystonia (OPD) is a form of focal dystonia characterized by involuntary movements that affect the facial, labial, and tongue muscles. OPD is frequently underrecognized and underdiagnosed, despite its relatively high prevalence among individuals with Parkinson’s disease (PD), including during the early stages of the disease. Limited awareness among both patients and healthcare providers can contribute to delays in diagnosis. We present the case of a 45-year-old Emirati male who presented with left-sided resting tremors followed by progressive tongue dystonia over four months. Clinical and radiological examination revealed him to have early-onset clinically established PD based on the Movement Disorder Society Clinical Diagnostic Criteria 2015. The patient was initiated on dopaminergic therapy, resulting in significant symptom improvement. Early recognition and timely intervention are essential, as they can substantially mitigate morbidity and enhance quality of life. This report examines the characteristics of dystonia, including its prevalence, etiologies, and clinical presentation within the context of early-onset PD, and outlines a tailored strategy to facilitate early diagnosis. The case highlights the importance of recognizing atypical features such as OPD in early-onset PD, which may present diagnostic challenges but respond well to timely treatment.

## Introduction

Both dystonia and parkinsonism are distinct syndromes with overlapping pathophysiology [1]. Early-onset Parkinson’s disease (EOPD) is characterized by an early onset with an age of 50 or below [2]. In addition, oropharyngeal dysphagia is also known to be a clinically significant symptom in patients with Parkinson’s disease (PD), which could be confused with oropharyngeal dystonia (OPD), leading to relative dysphagia [3]. Dystonia is a well-recognized feature of EOPD and is more frequently observed in these patients compared to those with late-onset PD. It may appear as an initial presenting symptom, often affecting the lower limbs, and could precede the development of classic parkinsonian signs such as tremor, rigidity, and bradykinesia [4].

Several genetic mutations have been associated with EOPD presenting with dystonic features. Approximately 3-5% of individuals with early-onset PD develop symptoms before the age of 40, often due to genetic mutations. Among these, Parkin gene mutations are the most frequently implicated in autosomal recessive early-onset PD [4]. Notably, mutations in the PARK7 and ATP1A3 genes have been implicated in familial and sporadic forms of early-onset parkinsonism with dystonia, highlighting the genetic basis of the disease in younger patients [4,5].

The pathophysiology of dystonia in EOPD primarily involves dysfunction within the basal ganglia circuits, which are essential for regulating motor control. This disruption results in abnormal signaling, leading to involuntary muscle contractions and posturing [5]. Clinically, patients may present with sustained muscle contractions, resulting in twisting movements or abnormal postures, particularly during action or at rest. In some cases, non-motor symptoms such as oculogyric crises, characterized by involuntary upward deviation of the eyes, may also be observed, further complicating the clinical picture [6].

## Case presentation

**Figure 1:**
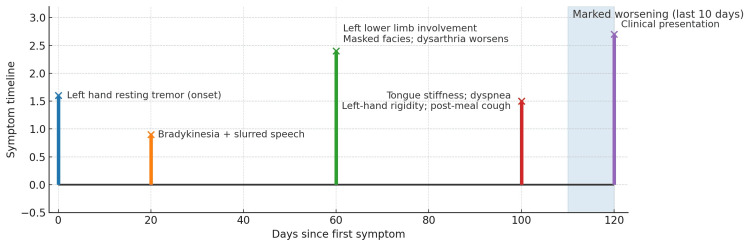
Timeline of symptoms leading to presentation

A middle-aged male in his 40s presented with a four-month history of progressive motor and oropharyngeal symptoms. His initial symptom was a resting tremor in the left hand, followed by slowing of movements (bradykinesia) and slurred speech. The symptoms gradually progressed to involve the left lower limb, and his family noted a masked facial expression and worsening dysarthria. More recently, he developed tongue stiffness with episodes of transient breathing difficulty, left hand rigidity, and coughing after meals, indicating evolving oropharyngeal dysfunction. These symptoms had worsened significantly over the past 10 days (Figure 1).

He visited multiple healthcare facilities without a definitive diagnosis. An MRI of the brain performed in another medical facility showed normal brain parenchyma. He had no significant past medical or surgical history, no allergies, no family history of neurological illnesses, and he leads a healthy lifestyle with no history of tobacco smoking or alcohol use. He is unmarried and employed in the police force.

On examination, the patient was alert and oriented with masked facies, slow speech, left-sided resting tremor, bradykinesia based on the Movement Disorder Society-Unified Parkinson’s Disease Rating (MDS-UPDRS) scale 10/40), rigidity based on the MDS-UPDRS scale 6/20), tongue and facial dystonia, and parkinsonian gait. He was started on Sinemet three times a day, with improvement in gait and OPD, though left-sided bradykinesia and rigidity persisted. His laboratory investigations were within normal limits (Table 1). No genetic testing was performed.

**Table 1:** Laboratory investigations ALP, alkaline phosphatase; ALT, alanine aminotransferase; AST, aspartate aminotransferase; eGFR, estimated glomerular filtration rate; LDH, lactate dehydrogenase; TSH, thyroid-stimulating hormone

Test	Result	Unit	Reference range
Bicarbonate (HCO₃)	23.8	mmol/L	22-28
Calcium	2.34	mmol/L	2.15-2.5
Corrected calcium	2.36	mmol/L	2.15-2.5
CK-MB (mass)	3.88	µg/L	≤5.0
Creatine kinase	75.9	U/L	40-171
Creatinine	68.1	µmol/L	80-115
eGFR	110	mL/min/1.73 m²	>60
CRP	0.8	mg/L	0.0-5.0
HbA1c	5.1	%	Normal: 4.0-5.6
LDH	112	U/L	135-225
Magnesium	0.761	mmol/L	0.66-1.07
Potassium	4.04	mmol/L	3.5-5.1
Sodium	134.5	mmol/L	136-145
Urea nitrogen	6.7	mmol/L	2.14-7.14
Inorganic phosphate	1.09	mmol/L	0.81-1.45
Total bilirubin	12.9	µmol/L	<21.0
Direct bilirubin	5.47	µmol/L	<7.0
ALP	62	U/L	40-129
ALT	57.5	U/L	<50
Albumin	38.9	g/L	35-50
AST	24.3	U/L	<50
Total protein	66.1	g/L	64-83
Chloride	101.9	mmol/L	98-107
Anion gap	12.65	mmol/L	10-20
TSH	2	uIU/mL	0.27-4.20
Troponin T	4.43	ng/L	<14

On follow-up one week post-discharge, he was conscious and oriented, with clear but hypophonic speech. There was mild improvement in persistent bradykinesia, more pronounced on the left (MDS-UPDRS bradykinesia score 8/40), and mild improvement in rigidity of the left upper limb (MDS-UPDRS rigidity score 4/20). He continued to exhibit a parkinsonian gait with loss of left arm swing. No tongue dystonia was observed at this visit.

## Discussion

Dystonia is a movement disorder characterized by sustained or intermittent involuntary muscle contractions that result in abnormal, repetitive movements [7]. When focal dystonia affects the orofacial and cervical regions, it may involve the muscles of the face, jaw, tongue, and neck, leading to significant functional impairment. A specific subtype of oromandibular dystonia (OMD) primarily affects the muscles of mastication, lips, and tongue. It may be task-specific or activity-related [7,8].

The precise pathophysiology of OMD remains poorly understood. However, it is believed to involve functional abnormalities in the subcortical forebrain motor circuits, including the basal ganglia, cerebellum, and brainstem, all regions implicated in motor control [6,7]. Disrupted sensorimotor integration, maladaptive neuroplasticity, and imbalances in neurotransmission, particularly involving dopamine and acetylcholine, are thought to contribute to symptom onset [6-8].

OMD may present as an isolated condition or in association with broader syndromes such as Meige syndrome, segmental dystonia, or generalized dystonia [7,8]. Rarely, it may progress to a dystonic storm, a life-threatening emergency characterized by generalized dystonia and autonomic dysfunction [7].

From a genetic standpoint, dystonia can represent a common final pathway for multiple inherited conditions. Examples include DYT1 mutations, Wilson’s disease, and cerebral palsy [7]. More recently, heterozygous mutations in the GBA gene, commonly associated with Gaucher disease, have been identified as risk factors for PD, particularly in early-onset cases. These mutations are also linked to non-motor symptoms and cognitive impairment [9]. Similarly, mutations in iPLA2β (PLA2G6), which encodes a calcium-independent phospholipase, have been implicated in autosomal recessive early-onset parkinsonism, often presenting with dystonia and oropharyngeal involvement [2,10].

In our patient, focal OMD was the initial symptom, preceding the development of classic features of advanced parkinsonism. Such atypical presentations may delay diagnosis, particularly in settings where access to neuroimaging and genetic testing is limited. This case highlights the importance of considering parkinsonian syndromes in the differential diagnosis of focal dystonia, especially in young patients [2,10].

Levodopa remains the mainstay of treatment for EOPD with dystonic features, typically improving both dystonia and parkinsonism symptoms [6]. However, long-term use can result in motor fluctuations and levodopa-induced dyskinesias, presenting challenges in younger individuals [6,9]. For refractory cases, deep brain stimulation targeting the globus pallidus internus or subthalamic nucleus has shown efficacy in reducing both dystonic and parkinsonian symptoms [6].

## Conclusions

This case illustrates the diagnostic challenge in EOPD patients with focal OMD as a presenting symptom. In younger patients, atypical presentations may be incorrectly identified as primary dystonia, leading to delays in accurate diagnosis and appropriate treatment. The progressive symptoms, satisfactory response to dopaminergic therapy, and genetic contribution emphasize the need for a high index of suspicion for parkinsonian-like syndromes in the context of isolated OPD. Early identification, prompt treatment, and genetic testing indication are essential for the management, outcomes, and knowledge of the clinical range of EOPD.
